# Risk factors for impaired maternal bonding when infants are 3 months old: a longitudinal population based study from Japan

**DOI:** 10.1186/s12888-019-2068-9

**Published:** 2019-03-08

**Authors:** Mami Nakano, Subina Upadhyaya, Roshan Chudal, Norbert Skokauskas, Terhi Luntamo, Andre Sourander, Hitoshi Kaneko

**Affiliations:** 1 ​Graduate School of Education and Human Development, Nagoya University, Furo-cho, Chikusa-ku, Nagoya, Japan; 2 Research Center for Child Psychiatry, University of Turku, Lemminkäisenkatu 3/Teutori 3rd Floor, 20014 Turku, Finland; 3 Centre for Child and Adolescent Mental Health and Child Protection, Faculty of Medicine, NTNU, NO-7491, Trondheim, Norway; 4 Psychological Support and Research Center for Human Development, Nagoya University, Furo-cho, Chikusa-ku, Nagoya, Japan

**Keywords:** Bonding, Postpartum depression, Population-based longitudinal study, Maternal negative feelings towards pregnancy, Primipara

## Abstract

**Background:**

Impaired maternal bonding has been associated with antenatal and postnatal factors, especially postpartum depression. Only a few population-based, longitudinal studies have examined the association between maternal depression and bonding in outside western countries. In addition, little is known about the association between psychosocial factors during pregnancy and impaired maternal bonding. The aim of this study was to investigate risk factors associated with impaired maternal bonding 3 months after delivery using Japanese population-based, longitudinal study from pregnancy period to 3 months after delivery.

**Methods:**

This study was performed at the public health care center in Hekinan city, Aichi prefecture, Japan. Mothers who participated the infant’s health check-up 3 months after delivery from July 2013 to Jun 2015 completed the Postpartum Bonding Questionnaire (PBQ) and the Edinburgh Postnatal Depression Scale (EPDS) 1 month after delivery. Information was also provided from home visit at 1 month after delivery, birth registration form, and pregnancy notification form. The study included 1060 mothers with a mean age of 29.90 years, who had given birth at a mean of 38.95 weeks.

**Results:**

Bivariate and multivariate logistic regression analyses were conducted to identify the association between antenatal and postnatal factors and impaired maternal bonding. The main findings were that maternal negative feelings about pregnancy (OR = 2.16, 95% CI = 1.02–4.56) and postpartum depression at 1 month after delivery (OR = 7.85, 95% CI = 3.44–17.90) were associated with higher levels of impaired maternal bonding 1 months after delivery. Mothers who had delivered their first child had increased odds of a moderate level of impaired maternal bonding 3 months after delivery (OR = 1.85, 95% CI = 1.22–2.81).

**Conclusions:**

The findings emphasize the importance of identifying mothers with depression and those with maternal negative feelings towards pregnancy to assess possible impaired maternal bonding.

## Background

The quality of the emotional tie between a mother and her baby, which was first described as bonding more than 40 years ago [1], is crucial for the infant’s survival and psychosocial development. Maternal bonding can start during the antenatal period [2] and is associated with postnatal bonding [3, 4]. Impaired maternal bonding can result in a higher risk of abusive parenting [5], poor mother-infant interaction [6] and children’s behavioral problems [7]. Studies have reported that the prevalence of impaired maternal bonding has ranged from 6 to 41% in clinical samples of the mother-infant dyads [8–13] to about 1% in the general population [9, 13, 14]. However, most of the previous research has been conducted in Western countries.

Impaired maternal bonding has been associated with a number of maternal risk factors, including both antenatal [3, 15] and postpartum depression [16, 17]. These results have been found in Western countries [3, 16, 17] and Asian countries [13], including Japan [18–22]. Impaired maternal bonding has also been associated with maternal anxiety [18], maternal insecure attachment [23], primiparity [24], poor family support [25], intimate partner violence [26], unintended pregnancy [27] and unplanned Cesarean section [28]. Furthermore, it has been associated with factors such as preterm birth [29], giving birth to a girl [13], and the infant’s sleep problems [7].

To our knowledge, most previous longitudinal studies investigating the risk factors for impaired maternal bonding did not collect data from the first trimester of pregnancy [13–19]. Although one study collected data from the first trimester [3], this information was collected for a proportion of participants retrospectively, which may pose a memory bias. As maternal bonding already starts to emerge early in pregnancy, it is of major importance to reliably identify early risk factors in order to be able to prevent the development of impaired maternal bonding. Additionally, in the majority of studies, maternal bonding has been measured during the first 2 months after giving birth [15, 18]. As it has been suggested that maternal bonding could be formed until 3 months after delivery [30], it is important to extend the assessment of bonding until then.

The present study addressed above highlighted knowledge gap by aiming to investigate the prenatal and postnatal risk factors associated with impaired maternal bonding 3 months after delivery. We hypothesized that postnatal depression symptoms and prenatal risk factors would be associated with impaired maternal bonding at this time point.

## Method

### Participants

This study was performed from July 2013 to June 2015 at the public health care centre in Hekinan city, Aichi prefecture, Japan. Hekinan city had a population of about 70, 000. The original cohort included 1163 mothers who participated the infant’s health check-up 3 months after delivery. Of note, 8.86% (*n* = 103) of mothers were excluded due to having twins (0.60%), not being Japanese (4.68%) and not completing the Postpartum Bonding Questionnaire (PBQ) [31, 32] (3.58%) 3 months after giving birth, resulting in total of 1060 mothers in the study.

The mean maternal age was 29.90 years (Standard Deviation (SD) 5.07, range 16–44 years) with the mean gestational age of 38.95 weeks (SD 1.37, range 33–41 weeks). Less than half of the participants (*n* = 425, 41.67%) were first-time mothers and just over half of the babies were male (*n* = 550, 52.78%).

### Procedure

This was a longitudinal, population-based study and the procedure was incorporated into the routine work carried out by the maternal and child health care service in Hekinan city, Aichi prefecture, Japan. In Japan, expecting mothers submit their pregnancy notification form to the public health care centre during the first trimester. After delivery, they have to submit the birth registration form to the municipal office within 14 days and this is automatically transferred to the public health care centre. The health condition of mother and infant 1 month after delivery is assessed by staff at public health care centre through home visit. Three to 4 months after delivery, the mothers visit the public health care centre for a checkup.

Information for the study was collected at four time points: the first trimester of pregnancy by pregnancy notification form, until 2 weeks after delivery by birth registration form, 1 month after delivery by home visiting, and 3 months after delivery by infant’s check-up.

### Measurements

#### Edinburgh postnatal depression scale (EPDS)

We used Edinburgh Postnatal Depression Scale (EPDS) [33, 34] to assess whether the mothers had postnatal depression one month after delivery. The EPDS is a self-rating scale and includes 10 items scored from 0 (no, not at all) to 3 (yes, all the time). Higher EPDS scores represent greater postnatal depression. The scale has a high internal consistency (Cronbach’s alpha 0.87) and good construct validity [33]. The Japanese version of the EPDS had good validity, and recommended a cut-off score of ≥9 to identify postpartum depression [34]. The sensitivity and specificity of the Japanese version of the EPDS have been reported to be 0.82 and 0.95, respectively [35]. In this study, 67 (6.45%) of the 1039 mothers who were assessed by the EPDS 1 months after delivery were identified as having postpartum depression.

#### Postpartum bonding questionnaire (PBQ)

We used the PBQ [31, 32] to assess maternal bonding during the infant health check-up 1 months after delivery. The original version of the PBQ [31] has 25 items with four factors. The Japanese version of the PBQ has 16 items with single factors with high reliability (Cronbach’s alpha 0.85) [32]. The back translation was confirmed by the original author of the tool [32]. The number of items used was different at the time of the study, as it was carried out while the Japanese version of the PBQ was still being developed. In this study, we used 15 items from the Japanese PBQ, as these were collected during the whole period of this study. Each item in this self-report scale is scored as 0 (never), 1 (rarely), 2 (sometimes), 3 (quite often), 4 (very often), and 5 (always), and a higher PBQ score means higher level of impaired maternal bonding. As the Japanese version of PBQ does not have validated cut-offs yet, we used data-driven cut-offs. A score above 24 (mean + 2 SD) was categorized as a high level of impaired maternal bonding, a score of between 17 and 23 (mean + 1 SD to mean + 2SD) was categorized as a moderate level of impaired maternal bonding and a score under 16 (mean + 1 SD) was categorized as a low level of impaired maternal bonding. Consequently, 35 (3.3%) of mothers were categorized as reporting a high level, 116 (10.9%) of mothers were categorized as reporting a moderate level, and 909 (85.8%) of mothers were categorized as reporting a low level of impaired maternal bonding. Although the Japanese version of the PBQ did not show normal distribution in Shapiro-Wilk test, normal distribution is not necessary for setting cut scores (e.g. [36]).

#### Additional study variables with single questions

##### Information from the first trimester of pregnancy

Single questions on the pregnancy notification form asked for the mother’s age (≤ 24, 25–30, 30–34 or ≥ 35 years), parity (primipara or multipara), maternal feelings towards pregnancy asking “How did you feel when you found out about this pregnancy?” (delighted or negative feelings i.e., unintended but happy, confused because unintended, or worried), maternal stress symptoms asking “Are you having symptoms such as sleeplessness, irritability, cry easily, lack of motivation etc continuously for 2 weeks during the last year?” (yes or no) and perceived mental illness before pregnancy asking “Have you suffered from a disease in the past or are currently getting treated for one (Disease name: mental illness (depression etc.))” (yes or no).

##### Information within 2weeks after delivery

The birth registration form included information about the infant’s sex (boy or girl) and birth-weight (≥2500 g or < 2500 g).

##### Information 3 months after delivery

Three months after delivery, during the infant checkup, the mothers were asked about details of the type of birth (vaginal delivery, Cesarean section, assisted delivery, i.e. vacuum extraction or forceps delivery), their feeding style (only breast-feeding, only bottle-feeding or combined breast- and bottle-feeding), maternal health after delivery asking “Do you have health problems after delivery?” (problems or no problems), maternal employment status during the antenatal period (employed or unemployed) and gestational age (≥ 37 weeks or < 37 weeks) (Table 1).

**Table 1:** Measurements across antenatal and postnatal period

Antenatal		Postnatal	
1. First trimester of pregnancy (Pregnancy notification form)	2. Within two weeks after delivery (Birth registration form)	3. One month after delivery (Home visiting)	4. Three months after delivery (Infant’s check-up)
1–1. Mother’s age	2–1. Infant’s sex	3–1. Depression (EPDS)	4–1. The type of birth
1–2. Parity	2–2. Birth-weight		4–2. Feeding style
1–3. Maternal feelings toward pregnancy			4–3. Maternal health after delivery
1–4. Maternal stress symptoms			4–4. Maternal employment status during the antenatal period
1–5. Perceived mental illness before pregnancy			4–5. Gestational age
			4–6. Maternal bonding (PBQ)

### Statistical analysis

Bivariate and multivariate logistic regression analyses were used to identify the associations between the antenatal and postnatal risk factors and impaired maternal bonding. The level of statistical significance used in the bivariate analysis was *p* < 0.10 to determine the variables for inclusion in the multivariate logistic regression analysis. The association with impaired maternal bonding to the exposure was reported as odds ratios (ORs) with 95% confidence intervals (95% CIs). In the logistic regression analysis, low level of impaired maternal bonding was set up as the reference. Moderate and high level of impaired maternal bonding were then compared to low level of impaired maternal bonding. The variables that were shown to be significant with impaired maternal bonding in the bivariate analysis (Table 2) were selected for the multivariate analysis (Table 3). Since post-partum depression is strongly associated with maternal bonding, we conducted separate multivariate analyses in two models. The first model included all other antenatal and postnatal variables without post-partum depression. The second model included all variables including post-partum depression. Although infant’s sex was not significantly associated with impaired maternal bonding in bivariate analysis, we included it in the multivariate analysis, because it is an important demographic variable and has been reported as an important risk factor for impaired maternal bonding especially in Asian culture [13]. The level of statistical significance was *p* < 0.05 for the multivariate analysis. All the statistical analyses were carried out in SPSS 23.0.

**Table 2:** Frequencies and univariable analysis of the independent variables during the antenatal and postnatal periods

Variable (*n* = responses to each question)	Level of impaired maternal bonding n (%)			Impaired maternal bonding	
				Moderate	High
	Low	Moderate	High	OR (95%CI)	OR (95%CI)
1–1. Mother’s age (Years) (*n* = 1040)					
< 24	146 (87.95)	17 (10.24)	3 (1.81)	0.97 (0.54–1.76)	0.65 (0.18–2.38)
25–30	350 (86.85)	42 (10.42)	11 (2.73)	Reference	Reference
31–34	222 (86.05)	23 (8.91)	13 (5.04)	0.86 (0.51–1.48)	1.86 (0.82–4.23)
> 35	176 (82.63)	30 (14.08)	7 (3.29)	1.42 (0.86–2.35)	1.27 (0.48–3.32)
1–2. Parity (*n* = 1020)					
Multipara	531 (89.24)	51 (8.57)	13 (2.18)	Reference	Reference
Primipara	345 (81.18)	59 (13.88)	21 (4.94)	1.78 (1.20–2.65)*	2.49 (1.23–5.03)*
1–3. Maternal feeling about pregnancy (*n* = 999)					
Delighted	624 (87.76)	70 (9.85)	17 (2.39)	Reference	Reference
Negative feeling (Unintended/Worried)	234 (81.25)	38 (13.19)	16 (5.56)	1.45 (0.95–2.21)+	2.51 (1.25–5.05)*
1–4. Maternal stress symptoms (*n* = 1036)					
No	851 (86.04)	110 (11.12)	28 (2.81)	Reference	Reference
Yes	39 (82.98)	2 (4.26)	6 (12.77)	0.40 (0.09–1.67)	4.68 (1.83–11.95)*
1–5. Perceived maternal mental illness before pregnancy (*n* = 1038)					
No	860 (86.26)	108 (10.83)	29 (2.91)	Reference	Reference
Yes	32 (78.05)	4 (9.76)	5 (12.20)	1.00 (0.35–2.87)	4.63 (1.68–12.76)*
2–1. Infant Sex (*n* = 1042)					
Boy	474 (86.18)	57 (10.36)	19 (3.45)	Reference	Reference
Girl	422 (85.77)	55 (11.18)	15 (3.05)	1.08 (0.73–1.61)	0.89 (0.45–1.77)
2–2. Birth weight (*n* = 1042)					
≥ 2500 g	835 (86.26)	104 (10.74)	29 (3.00)	Reference	Reference
< 2500 g	61 (82.43)	8 (10.81)	5 (6.76)	1.05 (0.49–2.26)	2.36 (0.88–6.31)+
3–1.Postpartum depression (*n* = 1039)					
No	848 (87.24)	104 (10.70)	20 (2.06)	Reference	Reference
Yes	44 (65.67)	8 (11.94)	15 (22.39)	1.48 (0.68–3.24)	14.46 (6.93–30.14)***
4–1. The type of birth (*n* = 1053)					
Vaginal delivery	653 (86.38)	81 (10.71)	22 (2.91)	Reference	Reference
Cesarean section	187 (84.23)	25 (11.26)	10 (4.50)	1.08 (0.67–1.74)	1.59 (0.74–3.41)
Assisted delivery	62 (82.67)	10 (13.33)	3 (4.00)	1.30 (0.64–2.64)	1.44 (0.42–4.93)
4–2. Feeding style (*n* = 1055)					
Only Breast feeding	587 (87.48)	68 (10.13)	16 (2.38)	Reference	Reference
Only Bottle feeding	122 (81.88)	21 (14.09)	6 (4.03)	1.49 (0.88–2.52)	1.80 (0.69–4.70)
Breast and bottle feeding	196 (83.40)	26 (11.06)	13 (5.53)	1.15 (0.71–1.85)	2.43 (1.15–5.15)*
4–3. Maternal health condition (*n* = 1056)					
Reported no problems	858 (85.71)	112 (11.19)	31 (3.10)	Reference	Reference
Reported problems	47 (85.45)	4 (7.27)	4 (7.27)	0.65 (0.23–1.84)	2.36 (0.80–6.95)
4–4. Employment status (*n* = 1059)					
Employed	482 (86.54)	56 (10.05)	19 (3.41)	Reference	Reference
Unemployed	426 (84.86)	60 (11.95)	16 (3.19)	0.95 (0.48–1.88)	1.21 (0.82–1.79)
4–5. Gestational age (*n* = 1059)					
≥ 37 weeks	876 (85.97)	111 (10.89)	32 (3.14)	Reference	Reference
< 37 weeks	32 (80.00)	5 (12.50)	3 (7.50)	1.23 (0.47–3.23)	2.57 (0.75–8.82)

*** < 0.0001, ** < 0.001, * < 0.05, + < 0.10

*NA* Not applicable

**Table 3:** Multivariate analysis with significant risk factors for impaired maternal bonding in univariable analysis, without adjusted depression and adjusted with depression

Variable	Multivariate analysis1) without adjusted depression	Multivariate analysis1) adjusted with depression		
	Moderate	High	Moderate	High
	OR (95%CI)	OR (95%CI)	OR (95%CI)	OR (95%CI)
1–2. Parity				
Multipara	Reference	Reference	Reference	Reference
Primipara	1.83 (1.22–2.76)*	2.47 (1.19–5.13)*	1.85 (1.22–2.81)*	1.97 (0.92–4.23)
1–3. Maternal feeling about pregnancy				
Delighted	Reference	Reference	Reference	Reference
Negative feeling (Unintended/worried)	1.53 (0.99–2.35)	2.40 (1.17–4.96)*	1.46 (0.94–2.28)	2.16 (1.02–4.56)*
1–4. Maternal stress symptoms				
No	Reference	Reference	Reference	Reference
Yes	0.36 (0.08–1.56)	2.58 (0.86–7.73)	0.22 (0.03–1.65)	1.85 (0.57–5.98)
1–5. Perceived maternal mental illness pregnancy before				
No	Reference	Reference	Reference	Reference
Yes	0.90 (0.26–3.13)	2.71 (0.82–8.92)	0.35 (0.05–2.68)	2.13 (0.59–7.71)
2–1. Infant Sex				
Boy	Reference	Reference	Reference	Reference
Girl	1.06 (0.71–1.59)	0.89 (0.44–1.84)	1.09 (0.72–1.65)	0.83 (0.39–1.77)
2–2. Birth weight				
≥ 2500 g	Reference	Reference	Reference	Reference
< 2500 g	1.06 (0.49–2.32)	1.96 (0.70–5.51)	0.99 (0.44–2.27)	1.95 (0.67–5.68)
3–1. Postpartum depression				
No			Reference	Reference
Yes			1.07 (0.43–2.65)	7.85 (3.44–17.90)***
4–2. Feeding style				
Only Breast feeding	Reference	Reference	Reference	Reference
Only Bottle feeding	1.34 (0.76–2.37)	1.13 (0.39–3.28)	1.21 (0.67–2.20)	0.98 (0.33–2.95)
Breast and bottle feeding	1.18 (0.72–1.94)	2.14 (0.97–4.71)	1.09 (0.65–1.82)	1.72 (0.75–3.94)

1) The multivariate analysis comprises variables that were shown to be significantly associated with impaired maternal bonding in the univariable analysis

*** < 0.0001, ** < 0.001, * < 0.05, + < 0.10

## Results

As shown in Table 2, in the bivariate analysis a number of factors during the antenatal and postnatal periods were associated with a high level of impaired maternal bonding. These were: maternal depression (OR = 14.46, 95% CI = 6.93–30.14), maternal stress symptoms (OR = 4.68, 95% CI = 1.83–11.95), perceived maternal mental illness before pregnancy (OR = 4.63, 95% CI = 1.68–12.76), maternal negative feelings towards pregnancy (OR = 2.51, 95% CI 1.25–5.05), being primipara (OR = 2.49, 95% CI = 1.23–5.03), combined breast- and bottle- feeding style (OR = 2.43, 95% CI = 1.15–5.15), and low birth-weight of the infant (OR = 2.36, 95% CI = 0.88–6.31). Moderate impairment was associated with being primipara (OR = 1.78, 95% CI = 1.20–2.65) and maternal negative feelings towards pregnancy (OR = 1.45, 95% CI = 0.95–2.21).

In multivariate analysis including depression (Table 3), maternal depression (OR = 7.85, 95% CI = 3.44–17.90) and maternal negative feelings towards pregnancy were associated with high level of impaired maternal bonding (OR = 2.16, 95% CI = 1.02–4.56). Primipara was associated with moderate level of impaired maternal bonding (OR = 1.85, 95% CI = 1.22–2.81).

We also conducted the multiple regression analyses with impaired maternal bonding as a continuous measure and confirmed that the same variables as in the logistic regression analysis, i.e. postpartum depression at 1 month after delivery (*β* = − 4.59, *p* < 0.01), maternal negative feelings towards pregnancy (*β* = − 1.58, *p* < 0.01), and primipara (*β* = − 2.52, *p* < 0.01) were significantly associated with impaired maternal bonding.

## Discussion

The primary aim of this study was to examine the risk factors associated with impaired maternal bonding using a large population-based sample in Japan. A number of important findings were identified. First, there was a strong association between postpartum depression 1 month after delivery and a high level of impaired maternal bonding 3 months after delivery. Second, maternal negative feelings towards pregnancy were associated with a high level of impaired maternal bonding, and becoming a mother for the first time was associated with a moderate level of impaired maternal bonding.

The finding that depressive symptoms during the postpartum period were associated with impaired maternal bonding at 3 months after delivery is in line with a previous Swedish population-based study that reported an association between postpartum depression 6 weeks after delivery and impaired maternal bonding 6 months after delivery [14]. Previous studies have also reported that impaired maternal bonding during the antenatal and postpartum periods was associated with postpartum depression [16, 19, 37, 38]. Previous longitudinal, population-based studies in non-western countries have not included psychosocial factors such as maternal stress symptoms, the history of mental illness and feeding style, when looking at the association between impaired maternal bonding and depression [13, 18, 19, 21, 26]. The present study added new knowledge by highlighting that depressive symptoms 1 month after delivery could have an effect on impaired maternal bonding later, even after controlled for these psychosocial factors.

The association between postpartum depression and impaired maternal bonding can be explained by several mechanisms, such as depressed mothers exhibited more negative feelings including irritation and decreased interest or joy in most activities [39]. Another explanation could be that depressed mothers display cognitive bias, such as negative perceptions of themselves and others, including their infant [40] and the future [41]. The features displayed by depressed mothers might promote negative maternal feelings, such as lack of concern or hostility towards the infant—features that have also been observed in mothers with impaired maternal bonding [42]. Our findings emphasize the importance of detecting maternal depression as soon as possible after delivery.

Maternal negative feelings towards pregnancy were independently associated with the high levels of impaired maternal bonding at 3 months after delivery, despite controlling for maternal depression. Previous studies have investigated the association between maternal feelings towards pregnancy or unintended pregnancy and impaired maternal bonding at one to 2 months after delivery [15, 18]. Our finding showed that maternal negative feelings towards pregnancy in first trimester of pregnancy could effect on maternal bonding 3 months after delivery. One possible explanation of the association between maternal negative feelings towards pregnancy and impaired maternal bonding could be that mothers who had negative feelings towards pregnancy might have also unwelcoming or ambivalent feelings towards their foetus. Another plausible explanation is that mothers with unintended pregnancies were more likely to have less support from their husband or partner [27]. Our finding suggests that health-service providers should pay close attention to mothers who express negative feelings after learning of their pregnancy, and should begin providing appropriate support from the early stages of pregnancy in order to prevent impaired maternal bonding.

Having first child was associated with only moderate level of impaired maternal bonding in the final model. One previous study reported that first-time mothers showed higher scores of impaired maternal bonding than mothers who already had other children [24]. The transition to motherhood can be a stressful event, characterized by emotional and physical changes as well as new responsibilities and demands [43]. Furthermore, mothers may be more anxious about taking care of their first-born child. Our finding suggests that early parenting advice would make capacity to form maternal bonding for first-time mothers.

In the bivariate analysis, mothers who experienced stress symptoms, had a history of mental illness, a low birth-weight infant, and who used combined breast- and bottle-feeding, had higher odds for impaired maternal bonding when their child was 3 months old. When these associations were controlled for the effect of other explanatory variables, they did not remain significant. However, these findings indicate that several other indicators for maternal wellbeing during pregnancy may be associated with impaired maternal bonding at a later stage. Moreover, there is a possibility that these antenatal risk factors might be associated with the mother’s mentalization, which is defined as the ability for one to understand their own mental state and those of others [44]. Mentalization plays an important role in the formation of the attachment between a mother and her infant [44], and it might also affect the mother’s emotions towards her foetus. The association between impaired maternal bonding and the mother’s mentalization ability deserves further research.

There were several limitations in this study that need to be considered. First, the participants were recruited from one Japanese city and 3.58% of the mothers were not included in the analysis because they did not want to reveal their bonding status or we did not have any information until their infants’ check-ups happened 3 months after delivery. It is possible that these mothers faced increased risks of impaired maternal bonding because they had not received services from the pregnancy period to after delivery from mother and child healthcare centres in the city. It is also possible that mothers did not want to answer, because they had impaired bonding with the infant. Second, due to the limited sample size, we could not investigate the association between impaired maternal bonding and unexpected problems, such as having a baby with a very low birth-weight or pregnancy complications. Third, information about impaired maternal bonding and postpartum depression were assessed using a self-rating scale and not by clinical interviews. However, the EPDS cut-off point has been shown to correlate with postpartum depression diagnoses using structured interviews [34]. Fourth, we used only 15 items of PBQ, while Japanese version of PBQ was validated with 16 items later. Fifth, it was not possible to adjust for residual confounding due to factors such as socioeconomic status, marital relationship, or infant’s temperament in this study. Finally, impaired maternal bonding was only assessed 3 months after delivery, so this study could not reveal any causal association between maternal depression and impaired maternal bonding. Having information maternal bonding at the mothers’ one-month follow-up visit could have strengthened the study.

The main strength of this study was the use of large, population-based sampling to investigate the risk factors of impaired maternal bonding. Of note, 91.14% of mothers 3 months after delivery living this city participated this study. This study adds to the current body of literature showing that postpartum depression can be one of the most important risk factors of impaired maternal bonding in both Japanese and Western societies. Second, the study design was longitudinal and included information collected during the first trimester of pregnancy. Thus, we found a number of antenatal factors, especially maternal negative feelings towards pregnancy, that were associated with impaired maternal bonding at 3 months after delivery.

## Conclusion

Our study reported that postpartum depression and maternal negative feelings towards pregnancy were independently associated with impaired maternal bonding. These findings have important clinical implications. Depressed mothers should be identified as soon as possible after delivery, in order to prevent impaired maternal bonding, and should be assessed to check how well they are bonding with their child. Furthermore, mothers who have negative feelings towards pregnancy should be followed carefully, as this could be an early sign of an increased risk of impaired maternal bonding, regardless of the maternal postpartum depressive symptoms that may surface later.

## Abbreviations

EPDS: Edinburgh Postnatal Depression Scale
PBQ: Postpartum Bonding Questionnaire
